# Reverse Trendelenburg Lithotomy with Certain Inclination Angles Reduces Stone Retropulsion during Ureteroscopic Lithotripsy for Proximal Ureteral Stone

**DOI:** 10.3390/jpm12122020

**Published:** 2022-12-07

**Authors:** Shihai Li, Jianchen Wu, Qiang Li, Jiawei Zhang

**Affiliations:** Department of Urology, First Hospital of Tsinghua University, Beijing 100016, China

**Keywords:** lithotripsy, proximal ureteral calculi, reverse Trendelenburg position, stone-free rate, ureteroscopy, urolithiasis

## Abstract

The objective of this study is to investigate how different inclination angles of reverse Trendelenburg lithotomy affect stone retropulsion and stone-free rates during ureteroscopic lithotripsy for proximal ureteral stones. Patients with proximal ureteral stones undergoing ureteroscopic lithotripsy in our institution between January 2019 and December 2020 were included according to predefined criteria. The rigid ureteroscope and Holmium: YAG laser were utilized to perform lithotripsy, and a stone basket was used to keep the stone in place and to avoid retropulsion. Before initiating lithotripsy, the upper part of the patient’s body was tilted up to establish a reverse Trendelenburg posture with appropriate inclination angles. To quantify the stone-free rate, computed tomography was used to evaluate the residual stones in the kidney one month following surgery. Patients’ clinical data were obtained retrospectively, including age, gender, the largest diameter of stone, stone density on computed tomography, and the distance between stone and ureteral pelvic junction, etc. Patients were divided into four groups based on the inclination angles of reverse Trendelenburg lithotomy: 0°, 10°, 20°, and 30°. The chi-square test was used to compare stone retropulsion and stone-free rates between groups. To discover possible determinants of the stone-free rate, logistic regression analyses were used. There were 189 patients that qualified. There were no differences in clinical characteristics between groups (*p* > 0.05). Multiple comparisons between groups revealed that the 20° and 30° groups had less retropulsion and a greater stone-free rate than the 0° and 10° groups (*p* < 0.05), whereas there were no significant differences in stone retropulsion or stone-free rates between the 20° and 30° groups or between the 0° and 10° groups (*p* > 0.05). The inclination angles as well as distance between the stone and ureteral pelvic junction were identified by using logistic regression analyses as the related factors for the stone-free rate. According to our results, the appropriate inclination angles of reverse Trendelenburg lithotomy during ureteroscopic lithotripsy for proximal ureteral stones would help preclude stone retropulsion and increase the stone-free rate.

## 1. Introduction

Because proximal ureteral calculi are closer to the pelvis than distal ureteral calculi, there is a higher chance that the stone or stone fragments will migrate into the pelvis during operations, compromising the efficiency of lithotripsy. Many studies have shown inconsistent findings about the stone-free rate (SFR) associated with different therapy regimens for proximal ureteral calculi. According to one study, ureteroscopic lithotripsy (URL) has a higher SFR than extracorporeal shock wave lithotripsy [1], but another system review suggests percutaneous nephrolithotomy over URL for the treatment of proximal ureteral stones [2]. Along with the development of ureteroscopy techniques and the innovation of lithotripsy devices, URL is becoming more commonly employed. The 2021 EAU guidelines on urolithiasis recommend URL as the first-line treatment for upper ureteral stones that are more than 1 cm in diameter, whereas ureteroscopy and extracorporeal shock wave lithotripsy are appropriate choices for proximal ureteral stones that are less than 1 cm in diameter.

As mentioned above, the retropulsion of stone fragments would compromise the SFR after the operation [3]. Even though the use of various anti-retropulsion devices significantly reduced the risk of retropulsion and increased SFR [4,5,6,7,8], stone fragment retropulsion is still unavoidable [9,10]. To treat residual stone fragments caused by retropulsion, a switch from using a rigid or semi-rigid ureteroscope to a flexible ureteroscope or additional procedures may be needed. Those measures may result in complications and an increase in healthcare costs. Consequently, additional methods for preventing stone migration to the pelvis during URL are warranted.

An in vitro study found that changing the inclination angle of the ureteral stone model during URL may decrease stone retropulsion [11]. They compared the stone retropulsion distance at the inclination angles of 0°, 10°, 20°, and 40°. The results showed that the higher the inclination angle, the less the retropulsion distance attained at a given time. The authors concluded that increasing the inclination angle of a patient may effectively preclude retropulsion when performing laser lithotripsy for ureteral stones. Thus, reverse Trendelenburg lithotomy may be a feasible method for avoiding stone retropulsion during URL. The in vivo viability of the reverse Trendelenburg posture for ureteroscopic lithotripsy has not yet been reported. In practical reality, would this position preclude stone retropulsion and boost SFR in the presence of several other confounders? The solutions are yet unknown. Gravity, in theory, works everywhere, so we sought to reduce the retropulsion of proximal ureteral stones in our clinical practice by inclining the upper body of patients in the lithotomy position; the inclination angles varied according to the sizes of the stones and their distance from the ureteral pelvic junction. Based on the real-world data collected retrospectively, we intended to evaluate the effect of reverse Trendelenburg lithotomy on stone retropulsion and SFR and to validate the feasibility of this posture in clinical practice.

## 2. Materials and Methods

### 2.1. Study Population

Patients who underwent retrograde URLs for single proximal ureteral stones in our institution between January 2019 and December 2020 were included according to the following criteria: patients who had a radiopaque proximal ureteral stone without ipsilateral kidney stone verified by non-contrast-enhanced computed tomography (NCCT); patients who did not have a ureteral stent in place prior to operation; patients who had residual fragments evaluated on day one after operation by kidney ureter bladder X-ray (KUB) and one month after operation by NCCT. Exclusion criteria were as follows: patients who had multiple ureteral stones or stones of >2 cm in diameter; patients who had ureteral deformity (stricture or congenital megaureter); patients who had stone impaction confirmed during operation. Due to the retrospective nature of the study, the fact that all procedures that were performed were part of the routine care, and the fact that all data were processed anonymously before being used, the informed consent and ethical review and approval were waived by the Ethics Committee of the First Hospital of Tsinghua University for this study.

### 2.2. Clinical Data

The following clinical data of patients were collected retrospectively: their gender, age, body mass index (BMI), operation duration, the largest diameter of the stone, the distance between the stone and ureteral pelvic junction (UPJ), the diameter of ureter above the stone, the Hounsfield Unite value (HU) of the stone on NCCT, side of the stone, the inclination angles of the upper part of patients’ bodies, and residual stones in the kidney one month after the operation. The proximal ureteral stone was characterized as a stone lying between UPJ and the upper edge of the sacroiliac joint. Residual stone fragments of >2 mm in diameter in the kidney were considered significant. Retropulsion was proven by the migration of stone particles of >2 mm into the kidney on KUB the following day after surgery. The HU value assigned to a stone was the mean value of the stone’s highest and lowest NCCT readings.

### 2.3. Operation Procedures

Following the completion of anesthesia, patients were positioned for lithotomy. A F12 urethral catheter was inserted into the bladder to drain urine and indwelled during the procedure. Continuous gravity-fed saline instillation was employed to maintain a clear vision, and the saline bag hung 100 cm above the bed. Under direct vision, a F8/9.8 Wolf rigid ureteroscope was inserted, and the orifice of the afflicted ureter was found. A guidewire with a flexible and hydrophilic tip (0.035 in/150 cm, Boston Scientific, Marlborough, MA, USA) was inserted into the ureter. Then, the ureteroscope was put forward along the guidewire. When the stone was clearly visible, it was secured in situ using a stone basket (3F/90 cm, uroVision, Rohrdorf, Germany). If the stone was too large to trap, the basket was inserted through the gap between the stone and the ureteral wall and opened just beyond the stone to stop retropulsion. To minimize retropulsion, before initiating lithotripsy, the upper part of the patient’s body was angled upward according to the size of the stone and the distance between stone and the UPJ. The stone was crushed with a 200 μm Holmium: YAG laser. The laser energy was adjusted to 0.5 J and the frequency was adjusted to 15 Hz to break the stone into fine fragments. Following lithotripsy, a thorough examination of the entire ureteral lumen was performed to rule out any residual fragments larger than 2 mm. Finally, an F6 ureteral stent was placed and left in place for three weeks after surgery; the F12 urethral catheter was changed to 16F and was removed the following day. The procedures were conducted by two well-trained attending doctors.

### 2.4. Patient Allocation

According to the inclination angles of the upper portion of the body after positioning the ureteroscope for lithotripsy, all patients were divided into four groups: 0°, 10°, 20°, and 30° group.

### 2.5. Statistic Methods

The SPSS software (version 26.0, IBM, Armonk, NY, USA) was used to conduct the statistical analysis. Continuous variables were presented as mean ± standard deviation (M ± SD), and categoric variables were presented as counts and percentages. The one-way ANOVA test was employed for comparisons between groups for variables with normality and variance homogeneity; otherwise, the Kruskal–Wallis H test was applied. The chi-square test was utilized to compare the difference between groups for categoric variables. The logistic regression was used to screen the associated variables for SFR. A *p* value of <0.05 was considered as statistically significant.

## 3. Results

### 3.1. Clinical Characteristics

Based on the inclusion and exclusion criteria, 189 patients were included in the study. Their clinical characteristics and results of comparison between groups are listed in Table 1. According to the Clavien–Dindo classification, most complications were classified as Grade I, such as fever, hematuria, and lumbar pain. Only urinary tract infections were classified as Grade II complications, and no complications of Grade III or higher ones were identified. No differences were revealed between the groups for all variables (*p* > 0.05).

### 3.2. Comparisons for Stone Retropulsion and SFR

The comparisons between groups for stone retropulsion and SFR one month after operation are presented in Table 2. According to the findings, stone retropulsion was significantly less in the 20°and 30° groups than in the 0°and 10° groups (*p* < 0.05). One month following surgery, the SFRs of the 20°and 30° groups were significantly higher than those of the 0°and 10° groups (*p* < 0.05). There was no difference between the 0° and 10° groups in terms of stone retropulsion and SFR (*p* > 0.05). The same result was also noted in the comparisons between the 20° group and the 30° group (*p* > 0.05).

### 3.3. Predictors for SFR

Based on their potential effects on SFR, the following variables were included in the logistic regression analysis to screen the significant factors for SFR: the largest diameter of the stone, the Hu value of the stone, the distance between the stone and UPJ, the diameter of ureter above the stone, operation duration, and inclination angles. The results are shown in Table 3. The inclination angles as well as the distance between the stone and UPJ were the related factors for SFR (*p* < 0.05).

## 4. Discussion

During the URL, many factors contribute to the retropulsion of stone fragments, which influences the final SFR. Several studies identified numerous elements that may have influences on stone retropulsion and SFR, including lithotripsy techniques, lithotripsy equipment, and laser device parameter settings [12,13,14,15]. Among those factors, the patient’s position during URL was one of importance.

The utility of the Trendelenburg position in URL for proximal ureteral stones has been explored in a prospective randomized controlled trial [16]. During the procedure, patients were positioned supine with their heads angled down below their feet at an angle of 30 degrees. According to the authors, this posture reduces the probability of stone fragments migrating to the inferior renal calyces. Consequently, the semi-rigid ureteroscope may be advanced farther into the renal pelvis to continue the laser lithotripsy procedure. Despite the substantial risk of stone migration, the authors concluded that this posture achieved a greater SFR than conventional lithotomy. Another randomized controlled trial came to the same outcome as the first [17]. Although this posture resulted in a higher SFR, it was associated with an increased risk of retropulsion inherently and was not recommended for routine use in the absence of a flexible ureteroscope for backup. Meanwhile, if the patient remains in this posture for an extended period of time, particularly for obese patients, it has detrimental influences on anesthesia management and hemodynamic stability [18]. In another research [19], the authors first placed the patient in supine lithotomy and then elevated the upper portion of the patient’s body at 40–45 degree; after that, they turned the patient’s affected side downward and the contralateral side upward to create a 30–45-degree angle between the level of the patient’s back and the level of the operation table. The authors concluded that this position significantly boosted SFR. However, it was overly complicated to implement. If the procedure lasted for a long period of time, it may cause harm to the lower portion of patient’s body.

Patients were classified into four groups in this retrospective investigation based on the inclination angles of their upper parts of bodies. No significant difference was noted between groups for the baseline characteristics, and our findings indicated that as inclination angles increased, the risk of stone retropulsion decreased during surgery and the SFR increased one month after surgery. In comparison to the 0°and 10° groups, the 20°and 30° groups showed decreased stone retropulsion and increased SFR (*p* < 0.05). An in vitro study achieved the same conclusion as ours [11]. The authors examined the distance of stone retropulsion at various slope degrees during laser lithotripsy using an in vitro ureteral stone model. The inclination angles were adjusted at 0°, 10°, 20°, and 40°. They discovered that retropulsion was reduced as the inclination angle of the saline-filled tube increased. The authors concluded that increasing the angle of a patient may effectively preclude retropulsion during URL. Together with our results, reverse Trendelenburg lithotomy is an appropriate technique for effectively preventing stone retropulsion during URL for upper ureteral stone. When the upper part of body is elevated, the gravity of stone combined with the instilled saline above the stone overcomes the retropulsion energy, thereby preventing stone retropulsion. This is a plausible explanation for this method.

Multiple comparisons between groups revealed no changes in stone retropulsion or SFR between the 20° and 30° groups or between the 0° and 10° groups. This finding indicated that only a specific scale of slope angles could reduce stone retropulsion; raising the inclination angle excessively or insufficiently would be ineffective. This discovery was the most significant finding of our retrospective investigation. Until now, no other research revealed results that were comparable. Regarding this phenomenon, we hypothesized that only when the inclination angle exceeded a certain value could sufficient gravity be generated by the stone and the instilled saline above the stone to counteract the retropulsion energy and so preclude the stone from retropulsion. However, when the inclination angle surpassed a particular limit, only fragments with small masses migrated upward to the kidney, and the SFR did not improve considerably after one month of spontaneous passage. A prospective randomized controlled trial will be required in future to establish an appropriate scale of inclination angles.

Additionally, the current study incorporated clinically significant variables in the logistic regression to screen for SFR-related factors. Compared with the indicator of 0°, an inclination slope of 20° or 30° could increase the SFR significantly. In contrast, the inclination angle of 10° would be ineffective. According to the findings, even when additional variables were present, inclination angles remained the significant predictors of SFR.

Due to the retrospective nature of this research, there are some limitations. The inclination angles were determined autonomously by the operator based on the stone’s size and the distance between the stone and the UPJ. It was impossible to regulate the increasing intervals to investigate the effects of continuous angles on stone retropulsion or SFR; a prospective trial would be needed to provide a more suitable scale with respect to inclination angles. A stone basket was used in the current study for each patient to assist in maintaining the position of the stone; investigating the difference in stone retropulsion and SFR between the group receiving reverse Trendelenburg lithotomy and the group receiving stone block devices is necessary. After patient allocation, the number of patients in each subgroup was relatively small, and a prospective cohort study is warranted to include a larger number of patients.

## 5. Conclusions

Based on the traditional lithotomy position used during URL with the Holmium: YAG laser for proximal ureteral stones, raising the upper part of the body to appropriate angles will decrease stone retropulsion and increase SFR significantly.

## Figures and Tables

**Table 1:** Summary of clinical characteristics and comparisons between groups with different inclination angles.

Variables		Inclination Angles			*p* Value
	0°	10°	20°	30°	
Patient number	85 (44.9)	38 (20.1)	43 (22.8)	23 (12.2)	
Gender					0.216
male	68 (80.0)	26 (68.4)	30 (69.8)	14 (60.9)	
female	17 (20.0)	12 (31.6)	13 (30.2)	9 (39.1)	
Age (year)	55 ± 13	54 ± 15	54 ± 12	54 ± 14	0.946
Stone largest diameter (mm)	9.3 ± 3.9	11 ± 4	10.6 ± 4.5	10.4 ± 4	0.109
Stone side					0.919
left	54 (63.5)	22 (57.9)	26 (60.5)	15 (65.2)	
right	31 (36.5)	16 (42.1)	17 (39.5)	8 (34.8)	
CT density of stone (Hu)	616 ± 194	683 ± 243	614 ± 206	723 ± 153	0.062
BMI	26 ± 4	26 ± 4	26 ± 4	28 ± 4	0.161
Distance from stone to UPJ (cm)	3.7 ± 1.0	3.4 ± 1.6	3.8 ± 1.1	3.5 ± 2.3	0.14
Ureter diameter above stone (mm)	9.2 ± 2.9	9.7 ± 2.5	8.5 ± 2.9	8.8 ± 3.0	0.09
Operation duration (min)	57 ± 28	66 ± 27	58 ± 28	64 ± 20	0.14
Complications					
Clavien grade I	6 (40.0)	3 (20.0)	4 (26.7)	2 (13.3)	0.940
Clavien grade II	4 (50.0)	1 (12.5)	2 (25.0)	1 (12.5)	1.000

Note: UPJ, ureteral pelvic junction; BMI, body mass index; CT, computed tomography. Data are expressed as *n* (%) or M ± SD.

**Table 2:** Comparisons of stone retropulsion and stone-free rates between groups.

		Inclination Angle				Total	χ^2^	*p* Value
		0°	10°	20°	30°			
Retropulsion	No	43 ^a^ (50.6)	20 ^a^ (52.6)	35 ^b^ (81.4)	21 ^b^ (91.3)	119	21.508	<0.01
	Yes	42 (49.4)	18 (47.4)	8 (18.6)	2 (8.7)	70		
Stone residual	No	50 ^a^ (58.8)	24 ^a^ (63.2)	39 ^b^ (90.7)	22 ^b^ (95.7)	135	22.328	<0.01
	Yes	35 (41.2)	14 (36.8)	4 (9.3)	1 (4.3)	54		
total		85	38	43	23	189		

Note: Data are expressed as *n* (%). The same superscript letter implies that there is no significant difference between groups, whereas a different superscript letter means that there is a significant difference between groups.

**Table 3:** Logistic regression analysis of related factors for stone-free rates one month after operation.

	Wald Value	*p* Value	OR	95% CI	
				Upper	Lower
Distance from stone to UPJ	10.276	0.001	0.592	0.429	0.815
Inclination angle *	18.901	0.000			
10°	0.941	0.332	0.654	0.277	1.542
20°	11.733	0.001	0.138	0.044	0.428
30°	9	0.003	0.038	0.005	0.323
CT density of stone	0.696	0.404	0.999	0.997	1.001
Operation duration	1.043	0.307	1.007	0.994	1.021
Stone largest diameter	1.633	0.201	0.943	0.863	1.032
Ureter diameter above stone	2.352	0.125	1.1	0.974	1.243

Note: UPJ, ureteral pelvic junction; CT, computed tomography; OR, odd ratio; CI, confidence interval. * 0° as an indicator.

## Data Availability

The data presented in this study are available on request from the corresponding author. The data are not publicly available due to hospital policy.

## References

[1] Rabani S.M., Moosavizadeh A. (2012). Management of Large Proximal Ureteral Stones: A Comparative Clinical Trial Between Transureteral Lithotripsy (TUL) and Shock Wave Lithotripsy (SWL). Nephrourol. Mon..

[2] Deng T., Chen Y., Liu B., Laguna M.P., de la Rosette J., Duan X., Wu W., Zeng G. (2019). Systematic review and cumulative analysis of the managements for proximal impacted ureteral stones. World J. Urol..

[3] Choi J.D., Seo S.I., Kwon J., Kim B.S. (2019). Laparoscopic Ureterolithotomy vs Ureteroscopic Lithotripsy for Large Ureteral Stones. JSLS.

[4] Elashry O.M., Tawfik A.M. (2012). Preventing stone retropulsion during intracorporeal lithotripsy. Nat. Rev. Urol..

[5] Ding H., Wang Z., Du W., Zhang H. (2012). NTrap in prevention of stone migration during ureteroscopic lithotripsy for proximal ureteral stones: A meta-analysis. J. Endourol..

[6] Fathelbab T.K., Abdelhamid A.M., Anwar A.Z.M., Galal E.M., El-Hawy M.M., Abdelgawad A.H., Tawfiek E.R. (2020). Prevention of stone retropulsion during ureteroscopy: Limitations in resources invites revival of old techniques. Arab. J. Urol..

[7] Jiang K., Male M., Yu X., Chen Z., Sun F., Yuan H. (2020). Efficacy and Safety of NTrap® Stone Entrapment and Extraction Device for Ureteroscopic Lithotripsy. Urol. J..

[8] Shabana W., Teleb M., Dawod T. (2015). Safety and efficacy of using the stone cone and an entrapment and extraction device in ureteroscopic lithotripsy for ureteric stones. Arab. J. Urol..

[9] Bagbanci S., Dadali M., Dadalı Y., Emir L., Gorgulu O., Karabulut A. (2017). Does a retropulsion prevention device equalize the surgical success of Ho:YAG laser and pneumatic lithotripters for upper ureteral stones? A prospective randomized study. Urolithiasis.

[10] Rane A., Bradoo A., Rao P., Shivde S., Elhilali M., Anidjar M., Pace K., JR D.A.H. (2010). The use of a novel reverse thermosensitive polymer to prevent ureteral stone retropulsion during intracorporeal lithotripsy: A randomized, controlled trial. J. Urol..

[11] Patel R.M., Walia A.S., Grohs E., Okhunov Z., Landman J., Clayman R.V. (2019). Effect of positioning on ureteric stone retropulsion: ‘gravity works’. BJU Int..

[12] Wollin D.A., Ackerman A., Yang C., Chen T., Simmons W.N., Preminger G.M., Lipkin M.E. (2017). Variable Pulse Duration From a New Holmium:YAG Laser: The Effect on Stone Comminution, Fiber Tip Degradation, and Retropulsion in a Dusting Model. Urology.

[13] Enikeev D., Grigoryan V., Fokin I., Morozov A., Taratkin M., Klimov R., Kozlov V., Gabdullina S., Glybochko P. (2021). Endoscopic lithotripsy with a SuperPulsed thulium-fiber laser for ureteral stones: A single-center experience. Int. J. Urol..

[14] Zehri A.A., Patel M., Adebayo P.B., Ali A. (2020). Inadvertent Stone Migration During Pneumatic Lithotripsy: Still a Conundrum in the 21st Century. Cureus.

[15] Finley D.S., Petersen J., Abdelshehid C., Ahlering M., Chou D., Borin J., Eichel L., McDougall E., Clayman R.V. (2005). Effect of holmium:YAG laser pulse width on lithotripsy retropulsion in vitro. J. Endourol..

[16] Pan J., Xue W., Xia L., Zhong H., Zhu Y., Du Z., Chen Q., Huang Y. (2014). Ureteroscopic lithotripsy in Trendelenburg position for proximal ureteral calculi: A prospective, randomized, comparative study. Int. Urol. Nephrol..

[17] Zhou R., Han C., Hao L., Chen B., Zang G., Fan T., Zhou J., Dong Y., Ma W., Pang K. (2018). Ureteroscopic lithotripsy in the Trendelenburg position for extracting obstructive upper ureteral obstruction stones: A prospective, randomized, comparative trial. Scand J. Urol..

[18] Arvizo C., Mehta S.T., Yunker A. (2018). Adverse events related to Trendelenburg position during laparoscopic surgery: Recommendations and review of the literature. Curr. Opin. Obstet. Gynecol..

[19] Zhang B., Lei Z.T., Shi Y.Q., Yang L., Liu W.G., Gao Q., Sima J. (2017). The efficacy of supine half-sitting lithotomy position in the treatment of upper ureteral calculi by surgery of ureteroscopic Holmium laser lithotripsy. Chin. J. Urol..

